# The inflammatory cellular constituents of foetal and infant leptomeninges: a survey of hospital-based autopsies without trauma

**DOI:** 10.1007/s00381-013-2348-5

**Published:** 2014-01-09

**Authors:** Esther Jack, Neil K. Fennelly, Terri Haddix

**Affiliations:** 1 Our Lady of Lourdes Hospital, Drogheda, Ireland; 2 Beaumont Hospital, Dublin 9, Ireland; 3 Forensic Analytical Sciences, Hayward, CA USA

**Keywords:** Leptomeninges, Infants, Inflammatory cells, Trauma, Shaken baby syndrome

## Abstract

**Objectives:**

Notwithstanding the lack of definitive evidence from studies conducted to date, inflammatory infiltrates and iron deposition in the leptomeninges are routinely used as forensic markers of traumatic brain injury. We investigated the presence of these forensic markers of trauma in neonates and infants, with the objective of determining their suitability for use in forensic cases.

**Methods:**

Leptomeninges derived from non-traumatic deaths were studied. Thirty-three cases were divided into groups 1 and 2, according to set age groups. Inflammatory cells and iron in these groups were quantified.

**Results:**

CD45, CD68 and CD163 positive inflammatory cells were identified in the leptomeninges of sections of the cerebellum, brain stem and cortex of all 33 cases of non-traumatic infant deaths surveyed in this study. There were no significant differences between the two groups. Iron was found in the leptomeninges in several cases, even those without recent haemorrhage. Overall within the two subgroups, the numbers of inflammatory cells and iron containing cells were not significantly different.

**Conclusion:**

These findings demonstrate that inflammatory cells and iron in the leptomeninges can be found in natural and non-traumatic conditions. Further, two cases with no reported neuropathology demonstrated the presence of inflammatory cells and iron. Thus, cautious interpretation of the presence of inflammatory cells and iron containing cells in forensic paediatric cases is recommended.

## Introduction

The leptomeninges are composed of the pia and arachnoid mater connected by strands termed arachnoid trabeculae. In the young, the leptomeninges are clear but become gradually thickened with age [1]. The leptomeninges and dura mater have been traditionally thought to contain few, if any, inflammatory cells, and any increase in cellularity is potentially equated to a pathologic condition, including inflicted trauma [1, 2]. At present, we do not know what constitutes ‘normal’ cellularity of infant leptomeninges and if inflammatory or iron containing cells should be present at all in non-forensic settings. However, when indeed present under suspicious circumstances, they are often linked to inflicted trauma, such as in cases of the ‘shaken baby syndrome’ [3].

In order to recognise and characterise the pathologic findings in infant brains, it is important to have an understanding of the normal constituents of the various intracranial compartments. While some studies in the past, largely in rodent pups [4], have sought to evaluate and characterise the leptomeningeal cellular constituents, up until now, a rigorous analysis of the inflammatory cellular constituents of the leptomeninges has not been performed in human late-foetal and infant brains. This characterisation will serve as a baseline for comparison with brains of similarly aged children in forensic settings.

Therefore, in addition to determining the inflammatory cellular composition and presence of iron in foetal and infant leptomeninges associated with natural disease processes and in the absence of physical trauma beyond that accompanying vaginal birth, this study aims to formulate a basis of comparison of leptomeningeal cellular constituents in forensic settings, based on rigorous histological analyses of hospital-derived autopsies.

## Materials and methods

### Subject selection

A total of 33 foetal and infant autopsies in which neuropathologic examinations had been performed at Stanford University Medical Center/Lucille Packard Children’s Hospital were identified utilising the department of pathology database program. The cases for study were chosen in concert with the attending neuropathologist responsible for rendering the original diagnoses. The criteria for study inclusion were cases between 2005 and 2008, the age bracket of late third trimester and 1 year of post-natal life, and availability of formalin-fixed paraffin-embedded tissue samples harvested from at least two different brain sites including the cerebral cortex, cerebellum and brainstem. There were three cases within these 33 total cases which did not conform to these criteria but were included.

### Leptomeningeal sample selection

Each sample slide was screened on microscopy by the attending neuropathologist, and only sections of brains reflecting a wide (≥5.0 mm) sampling of the leptomeninges were chosen.

### Sample fixing, staining and immunohistochemistry

Slides of routinely processed formalin-fixed, paraffin-embedded [5] sections in each case were prepared and stained with antibodies to CD45 (dilution 1:100), CD68 (dilution 1:100) and CD163 (dilution 1:200).

CD45, also known as leucocyte common antigen, is uniquely expressed on the surface of all leucocytes and their progenitor cells [6]—these include neutrophils, eosinophils, basophils, lymphocytes and monocytes. CD68 is expressed in monocytes and macrophages [7]. CD163 was used as an additional stain for cells of monocyte/macrophage lineage and microglia (although the density of microglia was not specifically evaluated in this study) [8].

Immunoperoxidase staining was performed, following microwave antigen retrieval in citrate buffer at pH6, on an automatic stainer (Dako Autostainer, Universal Staining System) [9]. Iron was detected in sections utilising the standard Perl’s staining method [10].

### Examination of samples

In order to reduce inter-observer variation, the number of variously immunoreactive or iron containing cells was quantified by a single observer and representative slides reviewed for accuracy by a second observer. At a microscopic magnification of 20x, immunoreactive cells within leptomeninges were counted and recorded.

As the length of leptomeninges evaluated varied between slides, the length of leptomeninges scored was measured in millimetres and results recorded as immunoreactive cells/millimetre. Only leptomeninges on gyral surfaces were scored as it was impossible to accurately measure the length of the leptomeninges in the sulci.

### Analysis of results

The samples were divided into infants who died beyond 33 post-natal days up to 1 year (group 1) and newborns who survived up to 33 days (group 2)—these represent pre- and post-natal leptomeninges [11].

The mean number of CD45, CD68 and CD163 positive immunoreactive cells/millimetre, occurring in the cerebellum, cortex and brain stem, was calculated for groups 1 and 2, respectively. Iron was recorded as being either present or absent. To test for significant differences between the numbers of immunoreactive cells between groups 1 and 2, an unpaired, two-tailed student’s *T* test was employed.

## Results

### Demographics

Of the 33 cases, 16 were male and 17 female. Thirteen were born via vaginal delivery and 19 via Caesarean section. The mode of delivery of one case was not available. Sixteen cases involved infants who died beyond the post-natal age of 33 days (group 1), and 17 cases represented either foetuses or newborns who survived up to 33 post-natal days (group 2). One child (number 7) who survived to 16 months of age was included in group 1. There were two cases (numbers 25 and 30) involving foetuses in the 26th and 28th weeks of gestation which were included in group 2.

The general autopsy and neuropathology findings of both groups overlapped, and these included congestive heart failure, Noonan’s syndrome, micrencephaly and pontosubicular neuronal necrosis (Tables 1 and 2).

**Table 1:** General autopsy and neuropathologic findings for infants with a post-natal age greater than 33 days (group 1)

Case no	General autopsy findings	Neuropathology findings
1	Chronic aspiration/pneumonitis with MOSF	Oedema with herniation; HIE
	Seizure disorder	
4	Noonan’s syndrome	Micrencephaly; pontosubicular neuronal necrosis
5	Trisomy 21 with CHD	Haemorrhagic infarct (left periventricular)
7	CHD	Micrencephaly
8	Interstitial pneumonitis (cultures negative)	PVL
9	Heterotaxy–asplenia syndrome	Micrencephaly; multiple cortical and WM infarcts
11	Congenital pulmonary malformation	Multifocal acute HI changes
12	Pulmonary hypoplasia	Remote and focal acute HI changes
14	None	Agenesis of CC
	(Brain only)	
17	CHD with infarction	None
22	Cardiomyopathy	Multiple cerebral infarctions
	Sepsis with MOSF	
23	Foetal hydrops	PVL; right occipital infarction
26	Noonan’s syndrome	Micrencephaly; hypomyelination; organising SAH, SDH
27	Liver failure	Metabolic astrogliosis; neuronal pyknosis; bilateral subdural membranes
28	RSV bronchiolitis	Remote occipital infarct; subacute EDH
	PNA	
33	CHD	Cystic infarct (frontal)

*CC* corpus callosum, *CHD* congenital heart disease, *EDH* epidural haemorrhage, *GM* germinal matrix, *HI* hypoxic/ischaemic, *HIE* hypoxic/ischaemic encephalopathy, *MOSF* multiple organ system failure, *PNA* pneumonia, *PVL* periventricular leukomalacia, *RSV* respiratory syncytial virus, *SAH* subarachnoid haemorrhage, *SDH* subdural haemorrhage, *WM* white matter

**Table 2:** General autopsy and neuropathologic findings for foetuses and infants with a post-gestational age up to 33 days (group 2)

Case no.	General autopsy findings	Neuropathologic findings
2	CHD	None
3	CHD	Micrencephaly;
		Arhinencephaly;
		Neuronal necrosis, subiculum
6	Harlequin ichthyosis	None
10	Diaphragmatic hernia Omphalocoele	PSNN
13	PNA (Strep)	Metabolic encephalopathy;
		Hydrocephalus;
		Partial agenesis of CC
15	CHD	Thoracic meningomyelocoele;
		Hydrocephalus; possible partial agenesis of CC
16	CHD	Organising SDH
18	Cardiomyopathy	Acute HI change (focal);
	DiGeorge syndrome	PSNN;
		Remote dural haemorrhage
19	Pulmonary HTN	PSNN
20	Foetal hydrops	GM haemorrhage extending to SA;
		Diffuse HIE
21	Tetralogy of Fallot	PSNN; BG infarct
24	CHD	Focal SAH;
		Small WM haemorrhages;
		PSNN
25	CHD	PSNN;
		Focal HI, cerebellum;
		PVL;
		Parietal infarct;
		Organising SDH, IVH, SAH
29	None (brain only)	Diffuse HIE
30	NEC (MOSF)	Meningoencephalitis;
		PSNN
31	Multiple congenital abnormalities including CHD	GM haemorrhage with IVH and SAH; PSNN;
		Arhinencephaly
32	Pulmonary hypoplasia/HTN	GM haemorrhage with IV and SAH; PSNN

*BG* basal ganglia, *CC* corpus callosum, *CHD* congenital heart disease, *GM* germinal matrix, *HI* hypoxic/ischaemic, *HIE* hypoxic/ischaemic encephalopathy, *HTN* hypertension, *IV* intraventricular, *MOSF* multiple organ system failure, *NEC* necrotizing enterocolitis, *PNA* pneumonia, *PVL* periventricular leukomalacia, *SA* subarachnoid, *SAH* subarachnoid haemorrhage, *SDH* subdural haemorrhage, *WM* white matter, *PSNN* pontosubicular neuronal necrosis

Table 3 demonstrates the number of slides from each brain region and the range of length, mean and standard deviation of associated leptomeninges scored per slide. Overall, 39 sites were sampled from cerebral cortices, 37 from brainstems and 30 from cerebella. These involved leptomeningeal lengths between 5 and 83 mm. Overall, no statistically significant differences between the mean number of immunoreactive cells in the cerebellum, brain stem and cortex were observed between groups 1 and 2 (Table 4).

**Table 3:** Site and range, mean and standard deviations of lengths of leptomeninges in all cases

Sites	No. of sites sampled	Leptomeningeal length (mm)	Mean (mm) +/− SD
Cerebral cortex	39	5−41	18.8 +/− 10.5
Brain stem	37	10−83	28.7 +/− 17.0
Cerebellum	30	10−47	20.3 +/− 11.1

**Table 4:** Test for statistically significant differences between the mean number of immunoreactive cells in the cerebellum, brain stem and cortex between groups 1 and 2

Comparison (group 1 vs. group 2)		*P*-value
Cerebellum	CD45	0.623
	CD68	0.235
	CD163	0.457
Brain stem	CD45	0.689
	CD68	0.337
	CD163	0.172
Cerebral cortex	CD45	0.247
	CD68	0.149
	CD163	0.204

### Group 1

**Fig. 1:**
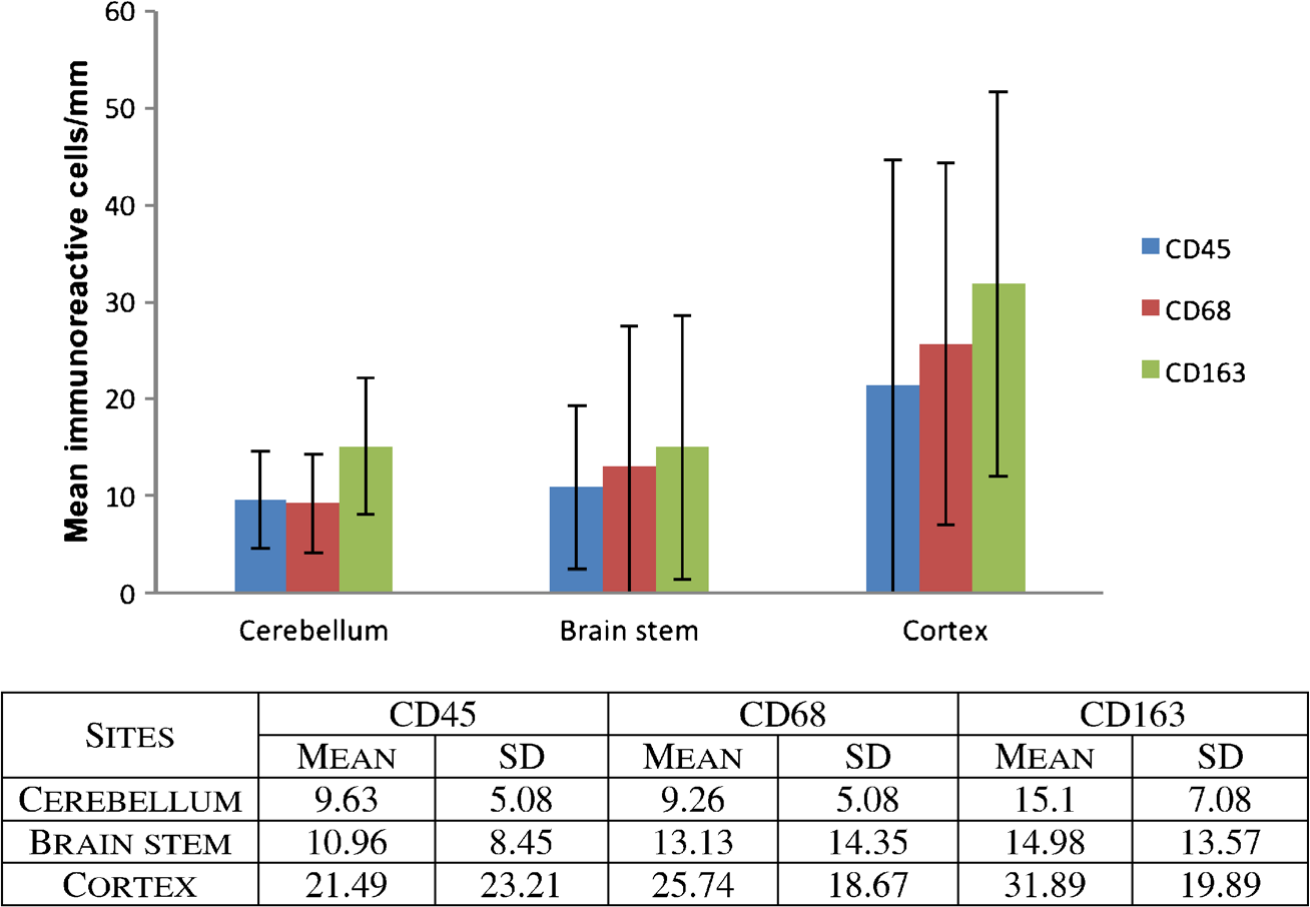
Mean immunoreactive cells/mm in cerebral cortex, brainstem and cerebellum (group 1)

Looking specifically at the cases in group 1 (Table 1 and Fig. 1), four had congenital heart disease (CHD) and two were diagnosed with Noonan’s syndrome. The mean number of CD45, CD68 and CD163 immunoreactive cells per mm of leptomeninges of the former group was 14.4, 17.5 and 17.9 cells/mm, respectively, and of the latter group 22.3, 18.6 and 21 cells/mm, respectively. Two cases in which sepsis or significant infection was documented (cases 22 and 8) had mean CD45, CD68 and CD163 counts of 19.3, 27.4 and 34.1 cells/mm, respectively. In group 1, 11 of 17 cases had some form of hypoxic/ischaemic event (pontosubicular neuronal necrosis, hypoxic-ischaemic encephalopathy, infarction, periventricular leukomalacia and hypoxic–ischaemic changes). The mean CD45, CD68 and CD163 positive cells/mm for this subgroup was 11.4, 15.5 and 19.0 cells/mm, respectively. Cases in the hypoxic/ischaemic subgroup incorporated cases in each of the other subgroups. Two cases had evidence of organising haemorrhages (epidural, subdural or subarachnoid; cases 26 and 28). The mean CD45, CD68 and CD163 cell counts/mm in this subgroup were 33, 35.8 and 40.7, respectively. These three cases had the highest mean CD45, CD68 and CD163 counts within group 1. These three cases also contained cells in the leptomeninges with stainable iron in at least one section. An additional 12 cases in group 1 demonstrated some degree of iron staining in at least one brain section.

Overall within group 1, the mean number of CD45, CD68 and CD163 cells in the cerebellum and brain stem was similar, with slightly higher numbers being observed in the cortex (Fig. 1)

### Group 2

**Fig. 2:**
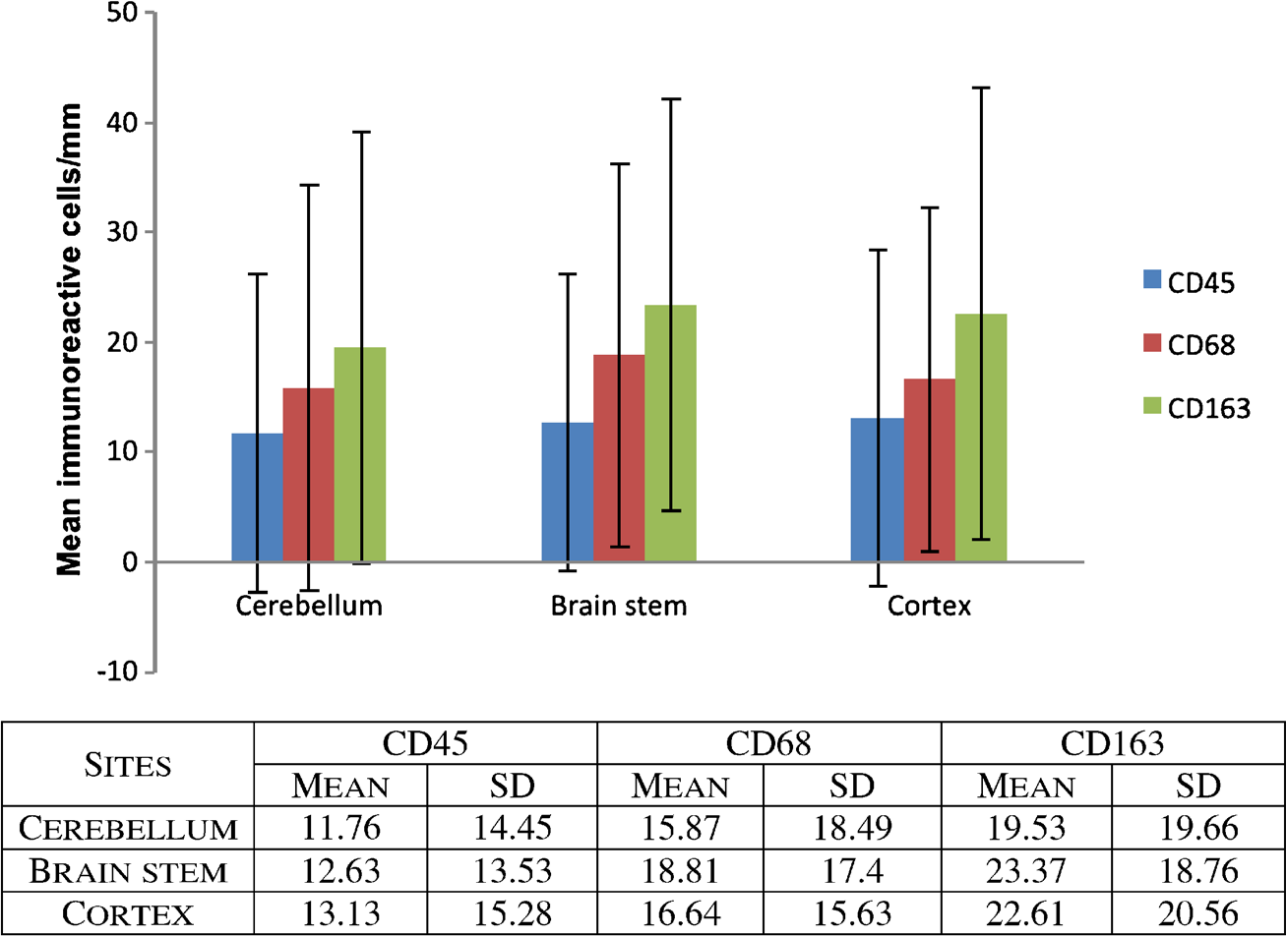
Mean immunoreactive cells/mm in cerebral cortex, brainstem and cerebellum (group 2)

Looking specifically at the cases in group 2 (Table 2 and Fig. 2), eight cases had CHD. The mean density of CD45, CD68 and CD163 immunoreactive cells/mm was 9.4, 16.6 and 23.2, respectively. Two cases in which sepsis or significant infection was documented had mean CD45, CD68 and CD163 counts of 31.8, 28 and 33 cells/mm, respectively. In group 2, 10 of 16 cases had some form of hypoxic/ischaemic event as noted above with CD45, CD68 and CD163 positive cells of 18, 20.5 and 24.6 cells/mm, respectively. Seven cases had some form of haemorrhage involving the dural surface or extending into the subarachnoid space. The number of CD45, CD68 and CD163 immunoreactive cells/mm in this subgroup was 13.5, 25.5 and 32.4 cells/mm, respectively. Five cases of seven in this subset with associated haemorrhage also had evidence of iron staining. Two cases with no reported neuropathologic findings had CD45, CD68 and CD163 counts of 5.9, 6.2 and 11.5 cells/mm, respectively. An additional eight cases in group 2 demonstrated some degree of iron staining in at least one brain section.

Overall within group 2, the mean number of CD45, CD68 and CD163 was similar across the locations sampled (Fig. 2).

### Cases without reported neuropathology

In three cases within both groups without neuropathologic abnormalities, all classes of inflammatory cells were found in the leptomeninges (case 17 of group 1; cases 2 and 6 of group 2).

### Iron findings

Of the 19 cases in which Caesarean sections were performed, 16 had positive iron findings, whereas 8 cases of the 13 vaginal births were positive for iron. Four cases of each mode of delivery reported haemorrhage-related neuropathologic diagnoses. Fifteen cases of Caesarean section births were associated with some form of hypoxic/ischaemic event in contrast to seven cases involving vaginal births.

## Discussion

**Fig. 3:**
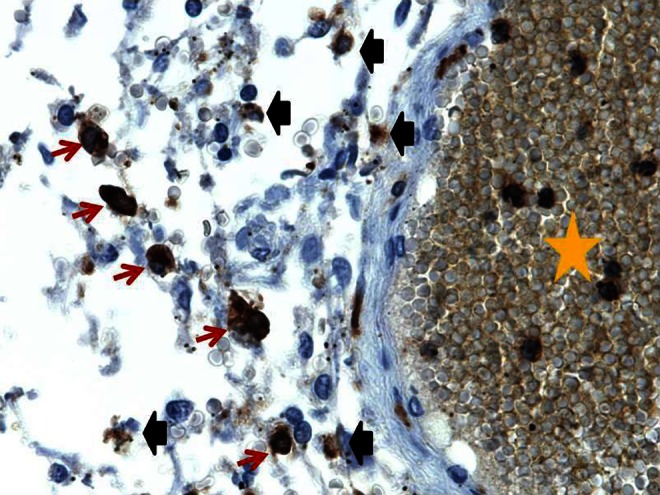
Representative case with CD45 immunostain (600×). Cells in the leptomeninges demonstrating appropriate positive staining are indicated with *red arrows*, and the cells and other constituents demonstrating non-specific staining are indicated with blunt *black arrows*. A vascular lumen containing some cells with appropriate staining is indicated by a *yellow star*. These intravascular cells were not included in counts. The *brown blush* elsewhere within the vessel represents additional non-specific staining

**Fig. 4:**
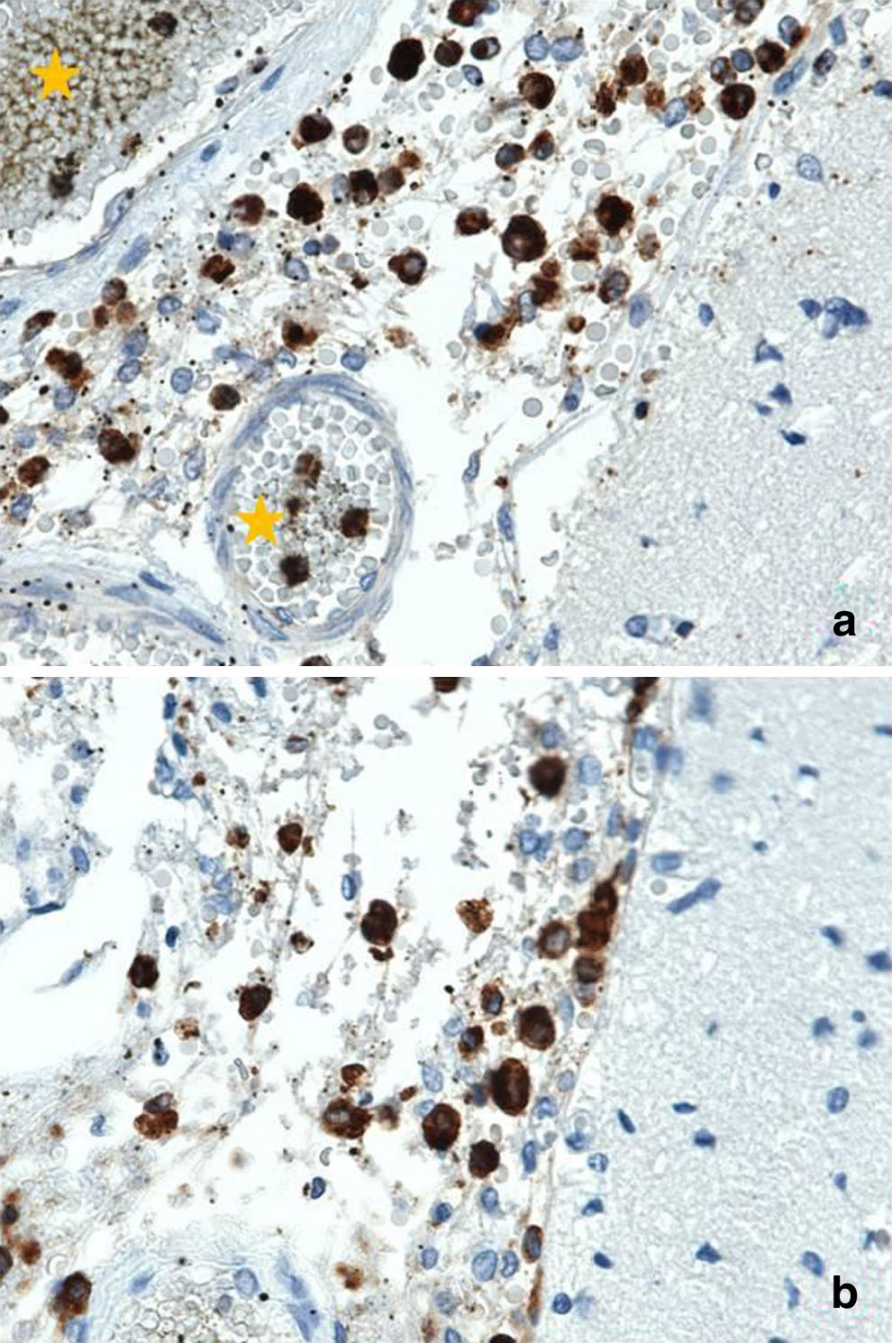
CD68 (panel **a**) and CD163 (panel **b**) immunostained sections (600×) from same case depicted in Fig. 3. In each panel, cerebral cortex is on the right side. In panel **a**, portions of two vessels (*yellow stars*) containing CD68 immunoreactive cells are also seen

In the current study, we found the presence of inflammatory cells in the leptomeninges, both overall and when segregated into two groups by age (foetal and early post-natal vs. infants beyond 33 days post-natal life) and by anatomic and neuropathologic conditions (Fig. 3). A notable finding is that even in foetuses and infants with no neuropathologic abnormalities, inflammatory cells and occasionally iron was identified in the leptomeninges. This is in contrast to the widely held belief that the leptomeninges should be largely devoid of inflammatory cells and iron in children with no reported neuropathology [12]. In addition, there was a positive correlation between the mean number of CD163 positive immunoreactive cells in cases with EDH, SDH or SAH pooled from both groups 1 and 2, when compared to those without these hemorrhages (mean immunoreactive CD163 cells/mm 30.34 vs. 15.12; *p* = 0.022). Interestingly, this correlation was not detected for the number of CD45 or CD68 positive cells and probably reflects the higher sensitivity of CD163 as a marker for monocytic cells (Fig. 4).

**Fig. 5:**
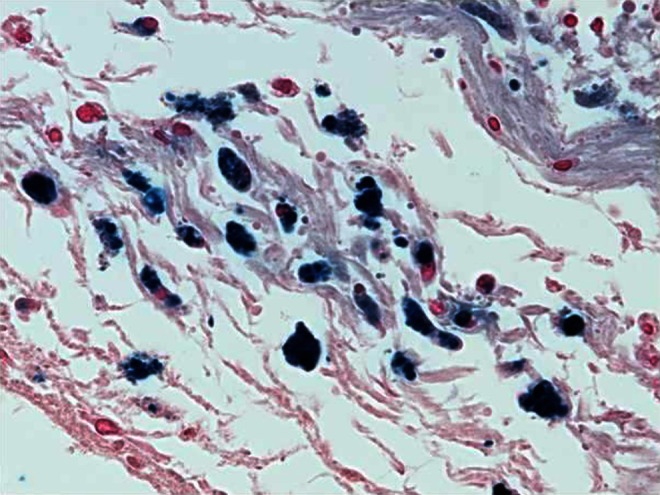
Example of iron staining in the leptomeninges (600×) in a different case than shown in Figs. 3 and 4. The leptomeninges are diagonally oriented downward from left to right and contain a large number of cells with an appropriate granular intracellular blue reaction product. In the upper right corner are some cells with non-specific staining

Caesarean section deliveries and vaginal births were associated with a variety of anatomic and neuropathologic diagnoses, with hypoxic/ischaemic events commonly found in both modes of delivery. Both modes of delivery were also associated with iron deposition in the leptomeninges and haemorrhage-related neuropathology (Fig. 5).

Accordingly, it appears that the presence of iron in the leptomeninges does not necessarily equate to traumatic haemorrhage but may be found in completely naturally occurring processes and occurs irrespective of the mode of delivery [2]. There also seemed to be no recurring pattern allowing us to associate the presence of iron to a single anatomic or neuropathologic diagnosis.

There are multiple mechanisms which allow the brain to sense inflammatory signals from systemic circulation [13], including interactions with circulating molecules in areas in the brain devoid of the brain–brain barrier. Microglial cells seem to migrate from the germinal matrix to the cortical layers. Early migration of microglia from the ventricular and meningeal surfaces has been observed as early as 4.5 weeks of gestation in humans [13].

The function of resting microglia under normal conditions is unclear. In pathologic conditions, these microglial cells are rapidly activated and proliferate. Animal studies have demonstrated that inflammation, either introduced systemically or within the brain, causes microglial activation along with cytokine release [14]. Accordingly, there is evidence to suggest that infection distant from the brain may damage developing foetal brain [13]. The activation of neuroinflammatory responses may also sensitise the brain to the damaging effects of other insults, such as hypoxia/ischaemia, and amplify the effects of the latter [14].

Thus, there are multiple possible reasons to account for the presence of inflammatory cells within the leptomeninges early in gestation in humans outside of inflicted trauma.

## Conclusion

We conclude that all patients with various natural disease processes in this hospital-based population had measureable numbers of CD45, CD68 and CD163 immunoreactive cells (and a subset had iron containing cells) in the leptomeninges overall and when segregated by age and anatomic and neuropathologic diagnoses. Although we studied a larger number of cases than the dura study by Croft et al. [2], our numbers of total cases are relatively small, particularly in consideration of the varied anatomic and neuropathologic diagnoses. Our cases were derived only from hospital autopsies, and these observations require comparison to actual forensic cases involving both traumatic injuries and natural disease processes. Clearly, the presence of inflammatory cells and iron in the leptomeninges can occur commonly, and in significant numbers, in non-traumatic neuropathologic conditions. As this study evaluated only non-forensic cases, we did not define a threshold level of inflammatory or iron containing cells above which a non-natural disease process (including inflicted injury) would be implicated. Thus, these findings support the recommendation of cautious interpretation of the findings of leptomeningeal inflammation and iron in forensic cases [3].
